# Post-COVID mRNA-vaccine IgG4 shift: worrisome?

**DOI:** 10.1128/msphere.00085-23

**Published:** 2023-05-16

**Authors:** Kamran Kadkhoda

**Affiliations:** 1 Immunopathology Laboratory, Robert J. Tomsich Pathology & Laboratory Medicine Institute, Cleveland Clinic, Cleveland, Ohio, USA; 2 Cleveland Clinic Lerner College of Medicine, Case Western Reserve University, Cleveland, Ohio, USA; Duke Human Vaccine Institute, Durham, North Carolina, USA

**Keywords:** COVID, vaccine, mRNA, IgG4

## Abstract

COVID-19 vaccines play a key role in ending the pandemic. Unraveling the immunological phenomena involved in offering protective immunity is the cornerstone of achieving such success. This perspective evaluates the possible mechanisms and implications of IgG4 production in response to mRNA-based COVID-19 vaccines.

Undoubtedly, the ongoing COVID-19 pandemic has stimulated unprecedented research on several aspects of this infectious disease. Despite the clear success in developing efficacious vaccines against COVID-19, the virus keeps evolving, and we must respond accordingly. As has always been the case in the vaccinology field, basic immunological studies are pivotal to unraveling the known unknowns and to fine-tune new vaccine development. As if the original antigenic sin and ongoing immune escape due to the emergence of new (sub)variants were not enough issues for long-term vaccine-induced protective immunity, it has recently been reported (1, 2) that repeated vaccination with mRNA-based vaccines caused a shift in anti-spike antibody repertoire toward IgG4 subclass, which is generally presumed to be anti-inflammatory. This effect, however, was not seen with adenovirus (Ad)-based vaccines. In this letter, I propose possible mechanisms underlying this fascinating finding.

It is known that mRNA-based vaccines lead to more spike antigen (Ag) shedding from cell membranes, even leading to a period of antigenemia, that is, the presence of cell-free circulating Ag (3, 4). This is associated with much stronger neutralizing antibody responses compared with Ad-based vaccines in which there is less shedding but more Ag presentation in the context of major histocompatibility complex I molecules leading to more robust CD8^+^ T cell responses. The soluble Ag has three different ways to reach the lymph nodes (Table 1): (i) multiple lymph nodes can be seeded during antigenemia, in which the Ag arrives in the lymph node via afferent blood vessels. After that, cognate naïve or memory B cells typically pick up the Ag and take it to germinal centers (GCs). They can internalize, process, and present to *T* cells or deliver the Ag to follicular dendritic cells (FDCs) in GC light zones; (ii) dendritic cells at the injection site can (macro)pinocytose the shed Ag, process, migrate to the ipsilateral lymph nodes (typically axillary ones when given in the deltoid muscle), and there, present to naïve *T* cells. The latter event, however, does not lead to loading FDCs with the native Ag; (iii) last but not least, soluble Ag that is shed from the deltoid muscle cell surfaces or released from disrupted cells due to local inflammatory response (mediated by pathogen- or danger-associated molecular patterns) into the extracellular environment. Then they are drained to the ipsilateral lymph nodes (typically axillary ones) via afferent lymphatics. After entering the subscapular sinus, Ags find their way to the deeper parts of the lymph nodes, and again cognate B cells pick them up and take them to GCs.

**TABLE 1:** Three possible mechanisms with which a given soluble antigen can reach a lymph node

Mechanism	Description
Antigenemia	Soluble Ag seeds multiple lymph nodes via their blood vessels. Cognate B cells pick up the Ag and take it to GCs where Ag is presented to *T* cells or delivered to FDCs.
Trojan horse	DCs at the injection site (macro)pinocytose the shed Ag, migrate to the regional draining lymph nodes and present to naïve *T* cells.
Lymph drainage	Soluble Ag in interstitium enters lymph, then it is drained to the regional draining lymph nodes via afferent lymphatics. After entering the subscapular sinus, Ags reach the deeper parts of the lymph nodes, and again cognate B cells take them to GCs.

Additionally, the processes mentioned above are accentuated when preexisting antibodies from previous infection or vaccination(s) bind the Ag. The resultant immune complex can also activate complement through classical pathway, producing local C3a and C5a. It is shown that follicular B cells express C3aR1 and C5aR1, which can signal these B cells to undergo class-switch recombination (CSR) in GCs in an autocrine fashion (5).

Even months after vaccination, the key to retaining GC reaction seems to rely on the immune complex reservoir on the surface of FDCs as produced through the mechanisms mentioned earlier. Maintaining and replenishing FDC surface reservoir is much more efficiently accomplished when complement components opsonize the Ag. It has recently been shown that spike Ag can directly activate complement via its lectin and alternative pathways (6, 7). Spike Ag, studded with complement degradation products such as C3b, iC3b, C3dg, and C3d, can more avidly bind B cells (that express complement receptors such as CD21). CSR requires activation-induced cytidine deaminase, which relies on CD21 (B cell) interaction with immune-complex bearing FDCs (8). The spatial organization of FDCs within the light zone of GCs is such that all FDC network captures and keep immune complexes. Still, the central, not the peripheral, FDCs function as long-term Ag reservoirs due to their higher expression of CD21 (9). The latter reservoir is replenished every time a booster is given, filling the memory B cell pool. The last requires rounds of “cyclic reentry” in the GCs, more somatic hypermutation, and CSR. The missing link to IgG4 CSR was possibly found when it was demonstrated that FDCs produced local interleukin-7 in GCs, which led *T* cells to favor CSR over IgG4 (10). IgG4 production typically occurs where there is long-term Ag exposure, such as in chronic parasitic infections or allergen immunotherapy (11 - 15) in the context of a conducive cytokine milieu, such as IL-4 and IL-10. It appears that when the FDC reservoir is “saturated” and memory cell frequency is adequate, introducing more Ag results in CSR to IgG4 to prevent making too many inflammatory antibodies, such as IgG1 and IgG3, both with efficient complement-fixing and FcR binding capabilities. Through Fab arm exchange and the so-called functional monovalency, IgG4 can block Ag surplus. So, the concern that mRNA-based vaccines trigger IgG4 after multiple boosters having implications for efficacy is not backed up by large-scale clinical trials; therefore, one cannot conclude that these vaccines are inferior to Ad-based ones merely due to a shift to IgG4. More importantly, it would be intuitive to speculate that class switching to IgG4 would happen where there is “enough” antigenic similarity between the incoming Ag and that of “complexed” on the surface of FDCs (from past exposures), obviating the need for undue rounds of cyclic reentry (original antigenic sin-like phenomenon). An emerging (sub)variant (be it from the virus or a vaccine), however, with relatively significant antigenic distance, would forgo IgG4 CSR and allow replenishing (reloading) of FDC surface repertoire and more efficient cyclic reentry leading at least to a reasonable cross-protection as expected to see from the boosters. This notion is further supported by lack of a large shift to IgG4 after repeated rounds of tetanus vaccination for whom no such antigenic variability is recognized (2). The shift to IgG4 also correlated with the overall observed increase in anti-spike IgG avidity, both strongly suggest repeated rounds of cyclic reentry (2).

At this evolutionary juncture, IgG4 is probably still figuring out its role in the immune response. The presence of IgG4, or lack thereof, does not necessarily insinuate protection from an infection or an exuberant immune response, a notion that is learned from chronic parasitic infections and IgG4-related diseases. Despite IgG4’s high levels in certain circumstances, the clinical picture remains not significantly different than when levels are within normal limits, as it appears that IgG4 is a by-product of another ongoing process. Undoubtedly, more in-depth research would shed light on the role of this new evolutionary member of the immune response.

## References

[1] Buhre JS , Pongracz T , Künsting I , Lixenfeld AS , Wang W , Nouta J , Lehrian S , Schmelter F , Lunding HB , Dühring L , Kern C , Petry J , Martin EL , Föh B , Steinhaus M , von Kopylow V , Sina C , Graf T , Rahmöller J , Wuhrer M , Ehlers M . 2022. MRNA vaccines against SARS-CoV-2 induce comparably low long-term IgG Fc galactosylation and sialylation levels but increasing long-term IgG4 responses compared to an adenovirus-based vaccine. Front Immunol 13:1020844. doi:10.3389/fimmu.2022.1020844 36713457 PMC9877300

[2] Irrgang P , Gerling J , Kocher K , Lapuente D , Steininger P , Habenicht K , Wytopil M , Beileke S , Schäfer S , Zhong J , Ssebyatika G , Krey T , Falcone V , Schülein C , Peter AS , Nganou-Makamdop K , Hengel H , Held J , Bogdan C , Überla K , Schober K , Winkler TH , Tenbusch M . 2023. Class switch toward Noninflammatory, spike-specific Igg4 antibodies after repeated SARS-Cov-2 mRNA vaccination. Sci Immunol 8:eade2798. doi:10.1126/sciimmunol.ade2798 36548397 PMC9847566

[3] Heinz FX , Stiasny K . 2021. Distinguishing features of current COVID-19 vaccines: knowns and unknowns of antigen presentation and modes of action. NPJ Vaccines 6:104. doi:10.1038/s41541-021-00369-6 34400651 PMC8368295

[4] Yonker LM , Swank Z , Bartsch YC , Burns MD , Kane A , Boribong BP , Davis JP , Loiselle M , Novak T , Senussi Y , Cheng C-A , Burgess E , Edlow AG , Chou J , Dionne A , Balaguru D , Lahoud-Rahme M , Arditi M , Julg B , Randolph AG , Alter G , Fasano A , Walt DR . 2023. Circulating spike protein detected in post-COVID-19 mRNA vaccine myocarditis. Circulation 147:867–876. doi:10.1161/CIRCULATIONAHA.122.061025 36597886 PMC10010667

[5] Paiano J , Harland M , Strainic MG , Nedrud J , Hussain W , Medof ME . 2019. Follicular B2 cell activation and class switch recombination depend on autocrine C3ar1/C5ar1 signaling in B2 cells. J Immunol 203:379–388. doi:10.4049/jimmunol.1900276 31217324 PMC7299189

[6] Ali YM , Ferrari M , Lynch NJ , Yaseen S , Dudler T , Gragerov S , Demopulos G , Heeney JL , Schwaeble WJ . 2021. Lectin pathway mediates complement activation by SARS-CoV-2 proteins. Front Immunol 12:714511. doi:10.3389/fimmu.2021.714511 34290717 PMC8287855

[7] Yu J , Yuan X , Chen H , Chaturvedi S , Braunstein EM , Brodsky RA . 2020. Direct activation of the alternative complement pathway by SARS-CoV-2 spike proteins is blocked by factor D inhibition. Blood 136:2080–2089. doi:10.1182/blood.2020008248 32877502 PMC7596849

[8] Aydar Y , Sukumar S , Szakal AK , Tew JG . 2005. The influence of immune complex-bearing follicular dendritic cells on the IgM response, Ig class switching, and production of high affinity IgG. J Immunol 174:5358–5366. doi:10.4049/jimmunol.174.9.5358 15843533

[9] Martinez-Riano A , Wang S , Boeing S , Minoughan S , Casal A , Spillane KM , Ludewig B , Tolar P . 2022 Long-term retention of antigens in germinal centres is controlled by the spatial organisation of the follicular dendritic cell network. bioRxiv. doi:10.1101/2022.09.06.506650 PMC7614842 37443283

[10] Jeannin P , Delneste Y , Lecoanet-Henchoz S , Gretener D , Bonnefoy JY . 1998. Interleukin-7 (IL-7) enhances class switching to IgE and IgG4 in the presence of T cells via IL-9 and SCD23. Blood 91:1355–1361. doi:10.1182/blood.V91.4.1355 9454766

[11] Adjobimey T , Hoerauf A . 2010. Induction of immunoglobulin G4 in human filariasis: an indicator of immunoregulation. Ann Trop Med Parasitol 104:455–464. doi:10.1179/136485910X12786389891407 20863434 PMC3065634

[12] Satitsuksanoa P , Angelina A , Palomares O , Akdis M . 2022. Mechanisms in AIT: insights 2021. Allergol Select 6:259–266. doi:10.5414/ALX02300E 36457721 PMC9707368

[13] Aalberse RC , Stapel SO , Schuurman J , Rispens T . 2009. Immunoglobulin G4: an odd antibody. Clin Exp Allergy 39:469–477. doi:10.1111/j.1365-2222.2009.03207.x 19222496

[14] Chung AW , Ghebremichael M , Robinson H , Brown E , Choi I , Lane S , Dugast A-S , Schoen MK , Rolland M , Suscovich TJ , Mahan AE , Liao L , Streeck H , Andrews C , Rerks-Ngarm S , Nitayaphan S , de Souza MS , Kaewkungwal J , Pitisuttithum P , Francis D , Michael NL , Kim JH , Bailey-Kellogg C , Ackerman ME , Alter G . 2014. Polyfunctional Fc-effector profiles mediated by IgG subclass selection distinguish RV144 and VAX003 vaccines. Sci Transl Med 6:228ra38. doi:10.1126/scitranslmed.3007736 24648341

[15] van der Neut Kolfschoten M , Schuurman J , Losen M , Bleeker WK , Martínez-Martínez P , Vermeulen E , den Bleker TH , Wiegman L , Vink T , Aarden LA , De Baets MH , van de Winkel JGJ , Aalberse RC , Parren P . 2007. Anti-inflammatory activity of human IgG4 antibodies by dynamic Fab arm exchange. Science 317:1554–1557. doi:10.1126/science.1144603 17872445

